# Investigating the Influence of Alkali Chloride Salts and Hydration on the Direct Air Capture Capacity of Polyethylenimine Films with Quartz Crystal Microbalance and Infrared Spectroscopy

**DOI:** 10.1021/acs.langmuir.6c00429

**Published:** 2026-06-03

**Authors:** Kayley Winata, Kayli Kuk, Christopher L. Soles, Avery E. Baumann

**Affiliations:** † 526980 Portola High School, Irvine, California 92618, United States; ‡ Northwest High School, Germantown, Maryland 20874, United States; § Materials Science and Engineering Division, 10833 National Institute of Standards and Technology, Gaithersburg, Maryland 20899, United States

## Abstract

With carbon dioxide (CO_2_) concentrations on the rise, direct air capture (DAC) technologies to remove carbon dioxide from the atmosphere are attracting increased attention. One popular approach is to use aminopolymer sorbents, such as polyethylenei mine (PEI), to absorb the CO_2_ and capture it through chemical reactions with the amine groups. In practice, the CO_2_ capture efficiency in aminopolymer sorbents strongly depends on the relative humidity, where the theoretical CO_2_ capture efficiency can change by 2-fold in going from a dry to a humid environment through the formation of either an ammonium carbamate or carbamic acid capture product, respectively. In this study, we utilize monovalent chloride salts with increasing cation size (LiCl, NaCl, KCl, or CsCl) to modulate water uptake and thus product formation, quantified using both gravimetric and spectroscopic methods. We utilize tandem quartz crystal microbalance with dissipation (QCM-D) and polarization modulation infrared reflection absorption spectroscopy (PM-IRRAS) to decouple the individual uptake of CO_2_ and H_2_O from the total sorption mass. The added salts were found to alter the H_2_O sorption levels of the PEI films, thereby impacting the CO_2_ uptake. We complement QCM-D/PM-IRRAS measurements using a high-throughput attenuated total reflectance Fourier transform infrared (ATR-FTIR) spectroscopy cell that conditions PEI films under varying relative humidities at a constant CO_2_ concentration. The carbamate/carbamic acid formation and H_2_O absorption were quantified by measuring the intensity of their characteristic vibrational signatures, informed by the QCM-D/PM-IRRAS experiments. Overall, we find that the addition of salt additives enables tuning of H_2_O and CO_2_ sorption at various humidity levels, with the identity of the salt cation directing the relative component uptake. These observations provide insight into the effectiveness of additives, especially salts, in modulating the CO_2_ uptake of aminopolymer sorbents being deployed in strategic carbon capture applications.

## Graphical abstract

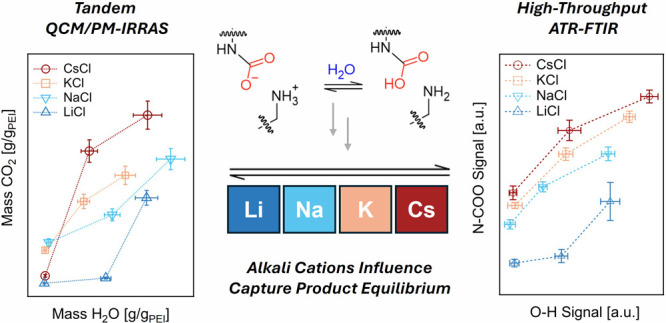

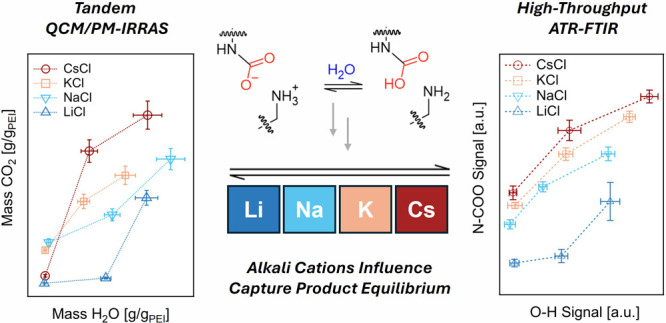

## Introduction

As global atmospheric CO_2_ concentrations have been steadily increasing, from preindustrial levels of approximately 280 ppm to over 400 ppm in 2025, the opportunity to recapture CO_2_ is becoming increasingly attractive.^1,2^ The direct air capture (DAC) of CO_2_ refers to technologies that remove CO_2_ from the atmosphere under ambient conditions. This means low concentrations of CO_2_ (≈400 ppm) as opposed to other methods of CO_2_ capture from pre- or postcombustion sources that operate at much higher CO_2_ concentrations (≈50,000 ppm or higher).^3^ Amine-rich sorbents, such as mesoporous silica impregnated with aminopolymers such as polyethylenimine (PEI), are suitable for DAC owing to their high density of primary, secondary, and tertiary amines as reported in a large volume of studies and comprehensive reviews.^4–8^ Many of these prior publications report that when CO_2_ is sorbed into a PEI film under dry conditions, the CO_2_ can react with the primary or secondary amines to form a zwitterion that quickly undergoes proton transfer to form one carbamate ion balanced by a neighboring ammonium ion, as shown in Scheme 1a. While the pairing of ammonium and carbamate is effective at capturing CO_2_, it also forms an ionic cross-link between the amines on neighboring PEI chain segments. We previously showed that this cross-linking can lead to a stiffening of the PEI films in the absence of a porous support that inhibits swelling and a subsequent reduction in the capture capacity.^9^ The stoichiometry of carbamate-ammonium pair formation dictates that it requires two amines to capture one CO_2_ molecule, resulting in an amine capture efficiency of 0.5. The presence of H_2_O, however, promotes the formation of a carbamic acid–water complex, which requires only a single primary or secondary amine to react with one CO_2_ molecule, increasing the theoretical amine CO_2_ capture efficiency from 0.5 to 1 (Scheme 1a).^8,10^ The carbamic acid route also does not create ionic cross-links and potentially preserves the softness and mobility of the PEI film.^11^ Furthermore, while we do not focus on regenerating the sorbent in this study, it has been previously shown that carbamic acid can be desorbed from amine sites at a lower temperature than the carbamate-ammonium capture product, which is ideal for preserving energy efficiency in DAC systems.^12–14^ Seeing an opportunity to exploit these different reaction mechanisms, we hypothesize that the ability to tune the H_2_O uptake of these PEI-based sorbents in different relative humidity environments may be a convenient handle to investigate water’s influence on CO_2_ capture. We pursue this notion by adding a series of alkali metal chloride salts to the aminopolymer film to untangle the complex interactions between H_2_O, CO_2_, the salt ions, and PEI.

**Scheme 1:**
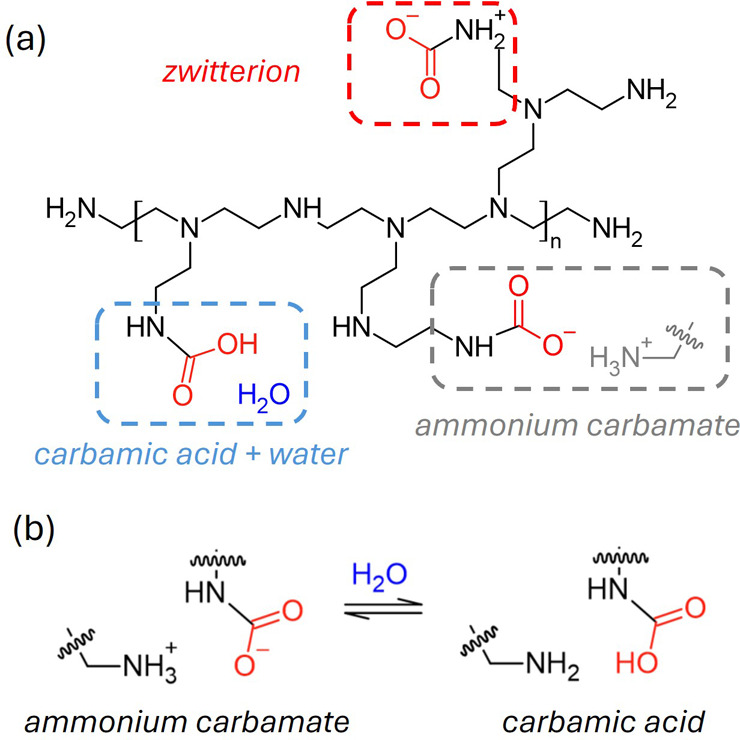
(a) Structure of PEI Showing Different Primary, Secondary, and Tertiary Amines; The CO_2_ Capture Products (Red) Are Shown as the Zwitterion Intermediate, Carbamate (Counterbalanced by Intermolecular Ionic Interaction Ammonium, Gray), and Carbamic Acid Formed in the Presence of Water (Blue); (b) The Intermolecular Ammonium Carbamate Complex Is in Equilibrium with Carbamic Acid Mediated by Water

The carbamate and carbamic acid capture products exist in equilibrium in the presence of H_2_O or protic solvents (Scheme 1b). A previous electrochemical study using small-molecule amines to shuttle CO_2_ to the electrode surface as carbamic acid found that the identity of salt cation, and not the anion, increases the equilibrium concentration of carbamate following the order, Li^+^ > Na^+^ > K^+^ in organic solution.^15^ The authors attribute this shift in the equilibrium from the carbamic acid to carbamate formation to the smaller cation, which leads to a stronger cation-carbamate ionic interaction. An additional investigation with both monovalent and divalent cations in solution suggests that added salts also disrupt the local hydrogen bonding networks and further shifts the carbamic acid equilibrium more toward the carbamate ion formation in an amine-CO_2_ adduct system.^16^ In another study investigating the effects of counterion identity on DAC in amino acid salt solutions, larger cations led to lower solution viscosities and enhanced CO_2_ sorption capacities.^17^ We hypothesize that the addition of Lewis acid cations, such as group 1 alkali metal salts, to the aminopolymer film may similarly shift the carbamic acid-to-carbamate equilibrium in PEI, influencing the multifaceted interactions between H_2_O, CO_2_, and amines by modulating H_2_O sorption and intermolecular interactions. Although this study focuses on PEI films without a porous support, numerous reports describe that water sorption in zeolites is influenced by addition of alkali salts where smaller cations (Li^+^ and Na^+^) coordinate water more strongly than larger cations (K^+^ and Cs^+^) owing to periodic trends of charge density and polarizability.^18,19^ Further, sorption can be gated by the cation identity due to size effects, where larger cations can limit pore accessibility and gate internal diffusion.^19–21^ These facets would need to be investigated if PEI with added salts were imbibed into porous support architectures.

Using a tandem quartz crystal microbalance with dissipation (QCM-D) and polarization modulation infrared reflective absorption spectroscopy (PM-IRRAS) instrument, we quantify the effects of DAC on the aminopolymer thin films with the added Li^+^, Na^+^, K^+^, and Cs^+^ chloride salts. Combined, these instruments allow the determination of specific component uptake (in this case, CO_2_ and H_2_O) as a function of dosing conditions, such as variations in humidity, and as a function film attributes, such as thickness, as shown in our prior reports for PEI films without the presence of a porous support host.^9,22,23^ The total mass change is measured by the QCM-D, while species-specific peaks in the PM-IRRA spectra can be used to separate the total mass uptake from the QCM-D into the individual amounts of CO_2_ and H_2_O. This allows us to explicitly examine the influence of alkali metal cations on the sorption behavior. Our previous studies utilizing the same measurement platform and PEI film geometry found that the CO_2__capture of PEI films thicker than 50 nm were limited by amine accessibility, while thinner films were limited by CO_2__availability.^23^ Furthermore, we found that the effect of humidity is more apparent in thicker films where water sorption leads to plasticization that increases amine accessibility, overcoming limitations above 40% relative humidity at 25 °C.^22^ We hypothesize that this RH-gated accessibility threshold in films greater than 50 nm can be lowered further via addition of salt additives.

The dissipation factor from the QCM-D also provides a measure of the mechanical stiffness of PEI films as they are exposed to various DAC atmospheres, providing insight into intermolecular interactions that are linked to sorbent performance. In our previous study, we observed a softening effect with H_2_O uptake relative to the dry film (H_2_O plasticized the dry PEI) but it had a subsequent stiffening effect upon addition of CO_2_ to the hydrated film, presumably due to the formation of ionic carbamate cross-links and film densification.^9^ We believe this stiffening impedes the subsequent diffusion of additional CO_2_ in the film and lowers the overall CO_2_ capture efficiency, leading to a lower number of primary and secondary amines being utilized for DAC. However, dosing the PEI sorbent with H_2_O concurrently with CO_2_ promotes the formation of carbamic acid, reversing the ionic cross-link formation, softening the film, and increasing amine efficiency of the capture process. While it is difficult to decouple these contributing factors, we can make inferences by comparing QCM-D results alongside our component uptake measurements for the PEI series with and without salt additives.

In addition to QCM-D/PM-IRRAS measurements, we include results from a custom-built high-throughput attenuated total reflectance Fourier transform infrared (ATR-FTIR) spectroscopy cell, integrated with H_2_O and CO_2_ gas dosing capabilities, that can measure up to 24 samples in a single dosing profile. This cell configuration is designed such that multiple samples are assessed under identical conditions and thus undergo the same conditioning procedures, enabling direct comparison for samples of different compositions and statistical analysis for replicate experiments. Because the ATR-FTIR cell only provides spectroscopic information, uptake values are reported on a relative scale, whereas the QCM-D/PM-IRRAS method gives absolute quantities. Taken together, these two instrument platforms provide clues to intermolecular interactions that arise from salt additives in PEI and describe the fundamental behavior of the amine sites, H_2_O, and CO_2_.

## Methodology

Certain equipment, instruments, software, or materials are identified in this paper to specify the experimental procedure adequately. Such identification is not intended to imply recommendation or endorsement of any product or service by the National Institute of Standards and Technology (NIST), nor is it intended to imply that the materials or equipment identified are necessarily the best available for the purpose.

### Materials

All materials were used as received unless otherwise stated. Hyperbranched PEI (molecular mass 25 kDa, Sigma-Aldrich) was dissolved in either ethanol or a 4:1 by volume ethanol:water mixture to prepare solutions for film casting. Alkali metal salts CsCl (Alfa-Aesar), KCl (Sigma-Aldrich), NaCl (Sigma-Aldrich), and LiCl (Beantown Chemical) were dissolved in the 4:1 ethanol:water solution containing PEI. All solutions show complete dissolution with no visible precipitation or suspension at room temperature.

### PEI Film Casting

A 20 mg/mL solution of 25 kDa PEI in a 4:1 ethanol to water (by volume) was prepared for film casting via spin coating, targeting a 100 nm thick film. To ensure consistency, the spin coating protocol was developed using polished Si wafers, rather than the QCM or ATR-FTIR substrates, to determine spin acceleration, speed, and drying time that yields the target thickness. The as-cast PEI film thickness was measured using a M-2000XI variable angle spectroscopic ellipsometer (J.A. Woollam Co., Inc., Lincoln, NE). The PEI solution containing no added salt solution was spin-coated atop a Si substrate and measured to be approximately 100 nm thick with less than ±5 nm in variation. PEI-salt solutions were cast in an identical manner as the neat PEI solution, following the same spin coating protocol, to ensure consistency in sample preparation. Salts were added in a 1:10 molar ratio of salt (LiCl, NaCl, KCl, or CsCl) to PEI to the initially prepared solution. Films were prepared in the same fashion for both QCM-D/PM-IRRAS experiments and high-throughput ATR-FTIR experiments.

### QCM-D/PM-IRRAS

Tandem quartz crystal microbalance with dissipation (QCM-D) and polarization modulation-infrared reflection-absorption spectroscopy (PM-IRRAS) measurements were performed using a custom QCM-D cell (AWSensors, Valencia, Spain) mounted within the PM-IRRAS accessory of a Nicolet iS50 FTIR spectrometer (Thermo Scientific, Waltham, MA). The QCM-D cell contains an enclosed environment that allows for controlled exposure to a desired feed gas manipulated by a series of mass flow controllers (Alicat Scientific, Tucson, AZ) described previously.^22^

Films were cast onto 1 in. (25.4 mm) diameter gold-coated AT-cut quartz crystals (Microvacuum Ltd.) with a resonant frequency of 5 MHz. Gold-coated crystals provided the IR reflective surface for PM-IRRAS measurements. Prior to use, the QCM-D crystals were cleaned by sonicating for 15 min in acetone and 15 min in isopropanol. The crystals were dried using compressed N_2_ and exposed to ultraviolet-ozone (UVO) for 15 min prior to testing. QCM-D measurements were performed with the clean crystal at 25 °C under 750 standard cubic centimeters per minute (SCCM) N_2_ to obtain each crystal’s baseline frequency values.

The coated QCM crystals were placed within the QCM cell and heated to 110 °C under 750 SCCM N_2_ flow to remove any adsorbed CO_2_ and water prior to measurements. Once the cell temperature reached 110 °C, PM-IRRAS confirmed that observable CO_2_ and water were desorbed. Then, the sample was cooled to 25 °C while maintaining a 750 SCCM N_2_ flow to allow the QCM frequency measurement to stabilize prior to exposure of the film to CO_2_ or H_2_O. The difference in frequency (Δ*F*) normalized by overtone (*n*) between the bare and PEI-coated crystal was converted into the mass of PEI by using the Sauerbrey equation.^24^ The overtone-normalized frequency shift, Δ*F*/*n*, and the dissipation factor, *D*, were recorded for the *n* = 3 overtone (15 MHz). QCM-D allows for the monitoring of the dissipation of the film, which can be used to comment on the change in mechanical stiffness of the films as a function of dosing condition. The dissipation of films with different added alkali chloride salts was measured throughout the experiments to measure changes in rigidity following exposure to water and CO_2_.

Three mass flow controllers were used to adjust the relative flow of N_2_ and CO_2_ into the cell, as well as the relative humidity via a water bubbler.^22^ One flow controller was for metering CO_2_ (400 ppm), while the other two flow controllers were used to vary humid and dry N_2_ balance. The two lines were connected to a 4-way switching valve (VICI Valco Instruments, Houston, TX), which led into the QCM-D cell. QCM-D and PM-IRRAS measurements were collected in N_2_ for 5 min prior to CO_2_/H_2_O exposure, after which CO_2_ was exposed for controlled time periods. The QCM-D/PM-IRRAS tests were performed for 3 h at a CO_2_ concentration of 400 ppm and a relative humidity (RH) that was stepped up every hour (approximately 0%, 20% and 40% RH) using a balance of dry N_2_.

For PM-IRRAS measurements, a wavelength of 2000 cm^–1^ was used as the center frequency of the photoelastic modulator. Each spectrum represents the average signal over a 60 s sampling time over a range of 1000 cm^–1^ to 4000 cm^–1^. The H_2_O and CO_2_ concentrations of the incoming gas feed were measured using a LiCOR Li-850 analyzer in-line prior to the sample cell.

### ATR-FTIR

High-throughput attenuated total reflectance Fourier transform infrared (ATR-FTIR) spectroscopy was utilized to monitor the formation of carbamate/carbamic acid and sorption of water in the dosed PEI films. High-throughput FTIR data was acquired using a Nicolet 6700 (Thermo Scientific) and an AutoATR accessory (PIKE Technologies). The AutoATR accessory accepts a 24-well plate, with each well containing an individual Si microgrooved wafer (groove angle 35°, JackfishSEC) that serves as the ATR crystal. The multiple wells of the high-throughput ATR-FTIR enable simultaneous monitoring of up to 24 samples, allowing for parallelized sorbent testing and minimized variability due to differences in experimental conditions such as room temperature and sample conditioning. Dosing campaigns were conducted with both randomly mixed arrays of the salt-containing films and arrays of single salt-containing films for this study. Individual ATR crystals were removed from the plate and coated with 100 nm PEI films via spin coating prior to testing.

A custom gas flow apparatus (PIKE Technologies) that attaches to the well plate assembly enables atmospheric control to the wells, controlled by a flow controller array (Alicat Scientific) mirroring that of the QCM-D/PM-IRRAS setup that meters a constant flow of 400 ppm of CO_2_ and a balance of dry and humidified N_2_ (via a bubbler) to the cell to achieve the desired RH. The H_2_O and CO_2_ concentrations of the incoming gas feed were measured using a LiCOR Li-850 analyzer in-line prior to the sampling cell. Prior to measurements, the 24-well plate containing the coated crystals was heated to 110 °C using a hot plate under 750 SCCM of N_2_, ensuring the removal of preadsorbed CO_2_ and H_2_O. Experiments were done in 3 h intervals, with relative humidity increasing stepwise at each hour (at roughly 0%, 20%, 45% RH steps) achieved by adjusting the flows of dry and humid N_2_ with a constant concentration of 400 ppm of CO_2_.

Spectra were exported from the proprietary OMNIC software (Thermo Scientific) for further analysis. For CO_2_ uptake analysis, the difference between the 1312 cm^–1^ peak intensity (attributed to the N-COO skeletal vibration of carbamate/carbamic acid) and a specified baseline intensity at 1200 cm^–1^ provides an estimate of CO_2_ adsorption. The water sorption is estimated by taking the difference between the intensity at 3212 cm^–1^, a shoulder of the O–H stretching vibration band, and the specified baseline intensity at 3990 cm^–1^. All data processing in this manner was conducted using a Python script on the individual exported spectra collected at each time point. Spectra were collected in 2 min intervals. Data from multiple experiments was averaged and initialized using the intensity before dosing with CO_2_ or H_2_O, which was set to 0. This approach allowed for direct comparison of samples across cells with varying optical throughput relative to a blank reference cell. Reported error bars correspond to one standard deviation from the mean, representing the experimental uncertainty of the measurements.

Data collection and sample handling for each instrument differed depending on the capabilities of the platforms, summarized as follows. The QCM-D/PM-IRRAS had a higher sampling interval (1 min) than the ATR-FTIR (2 min) for spectroscopic data. QCM-D experiments were done on a single sample, while high-throughput ATR-FTIR allowed for simultaneous measurements of multiple samples. Though dosed with equivalent concentrations and amounts of the respective gases as the ATR-FTIR, there are slight differences in relative humidities used in the experiments for the QCM-D/PM-IRRAS (max 40% RH vs 45% RH for ATR-FTIR) due to daily variations in room temperature at the time of collection. Furthermore, the QCM-D cell is maintained such that the experimental temperature is held precisely at 25 °C, where the ATR-FTIR cell does not have temperature control capabilities, resulting in measurements at ambient temperature (23.5 to 24.5 °C). Future efforts could be made to mitigate these potential sources of error with more extensive instrumentation design.

## Results and Discussion

### QCM-D/PM-IRRAS

We utilize a tandem QCM-D/PM-IRRAS instrument to simultaneously measure the total mass uptake of H_2_O and/or CO_2_ and corresponding concomitant changes in infrared spectra that can be used to speciate the individual fractions of CO_2_ and H_2_O in the total mass uptake. Prior to multicomponent dosing experiments, the relationship between carbamate intensity measured by PM-IRRAS and mass change measured by QCM- D is discerned by exposing the PEI film to dry 400 ppm of CO_2_. The slower infrared spectra are collected over 1 min intervals, while QCM-D data can be collected at higher frequencies (here, ≈1 Hz). The relationship between mass uptake and the intensity of the carbamate absorption peak in the PM-IRRAS spectra under these dry conditions can then be used as a calibration to estimate the individual component uptake (CO_2_ and H_2_O) in mixed-gas dosing experiments. The process for estimating these individual components has been outlined in our previous publications.^9,22,23^ but is reproduced here for clarity. The partial uptake mass of CO_2_ (*M*_pCO_2__) in mixed-gas dosing experiments is determined using the relationship between carbamate peak intensity at 1312 cm^–1^, highlighted in the spectra in Figure 1a, and total mass uptake (*M*_tot_) in a single component dosing experiment conducted at 400 ppm of CO_2_ in N_2_ balance. The 1312 cm^–1^ peak represents the N-COO skeletal vibration and therefore accurately reflects the overall CO_2_ uptake, regardless of whether it follows the carbamate or carbamic acid pathways. This yields a constant term that can then be multiplied by the change in carbamate/carbamic acid signal (relative to a background intensity arbitrarily taken at 1200 cm^–1^, a flat region of the spectra) measured by PM-IRRAS as described in eq 1:

1 $${\left(M_{p{\mathrm{CO}}_{2}}\right)}_{{\mathrm{CO}}_{2}+H_{2}O}=[{\left(\frac{M_{\mathrm{tot}}}{I_{1312}-I_{1200}}\right)}_{{\mathrm{CO}}_{2}}\times {\left(I_{1312}-I_{1200}\right)}_{{\mathrm{CO}}_{2}+H_{2}O}]$$

**Figure 1:**
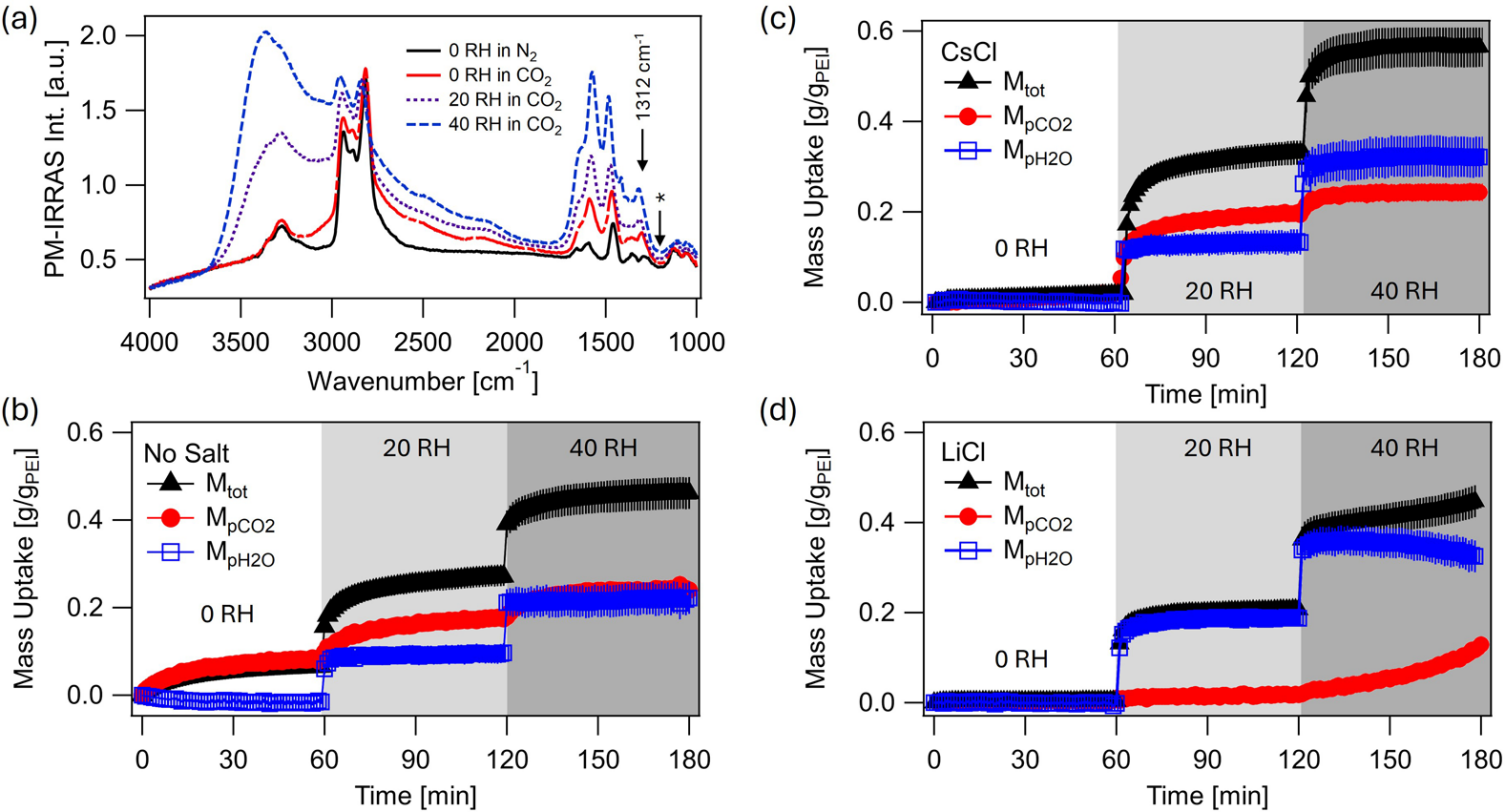
(a) PM-IRRA spectra of 100 nm PEI film with no salt added after 60 min of exposure to 400 ppm of CO_2_ at the specified humidity (0 RH, 20 RH, 40 RH) compared to the film conditioned under dry N_2_. *M*_pCO_2__ is derived from the PM-IRRAS signal at 1312 cm^–1^, indicated by the arrow in the spectra, and normalized to the baseline intensity at 1200 cm^–1^ (denoted by *). Tandem QCM/PM-IRRAS enables specific component uptake calculated using the CO_2_ signal for 100 nm films of PEI with (b) no salt, (c) CsCl, and (d) LiCl added. Error in *M*_tot_ and *M*_pCO_2__ is understood to be 8% of the value based on prior studies, error is propagated for the calculation of *M*_pH_2_O_. Lines in (b–d) are to guide the eye. The profiles for NaCl and KCl are included in the Supporting Information file.

The partial mass of water (*M*_pH_2_O_) is then derived by subtracting the calculated *M*_pCO_2__ from the *M*_tot_ measured in the mixed-gas experiment, as shown in eq 2:

2 $${\left(M_{pH_{2}O}\right)}_{{\mathrm{CO}}_{2}+H_{2}O}={\left(M_{\mathrm{tot}}\right)}_{{\mathrm{CO}}_{2}+H_{2}O}-{\left(M_{p{\mathrm{CO}}_{2}}\right)}_{{\mathrm{CO}}_{2}+H_{2}O}$$

The quantities *M*_tot_, *M*_pCO_2__, and *M*_pH_2_O_ are plotted as a function of time in Figure 1b–d for PEI films with no salt, with CsCl, and with LiCl over the course of three 60 min sorption steps occurring in atmospheres of 400 ppm of CO_2_ in dry (RH = 0.0 ± 0.3%), RH = 20 ± 2%, or RH = 40 ± 4% conditions. We select these RH values to best understand the interplay between PEI, water, salt, and CO_2_ (even though they may not represent an optimized condition for practical DAC application) based on findings from our previous study that the role of humidity is most apparent in these steps.^22^ Our previous investigation did include sorption results up to 80% RH, but the amine accessibility limiting capture in 100 nm films appeared to be overcome at 40% RH.^22^ The results for all samples, including the films containing KCl and NaCl additives, are included in Figure S1. All films are conditioned under dry N_2_ prior to exposure to CO_2_ and follow a profile where the RH is increasing as noted. We estimate the uncertainty of *M*_tot_ and *M*_pCO_2__ obtained from QCM-D/PM-IRRAS measurements to be within 8% of the measured value based on the highest variance in replicate experiments reported in our previous publication.^22^ The PEI with no salt exhibited a CO_2_ uptake profile that plateaued during the 1 h exposed to the differing humidities, indicating the film was approaching equilibrium. In contrast, we observed an increasing slope for the partial CO_2_ uptake graphs during the 1 h intervals for some of the PEI with salt samples, particularly LiCl at high humidity, which indicates that the equilibrium CO_2_ capacity has not been reached within the 1 h interval. We note that true equilibrium is not reached within 1 h for any of the films in any of the dosing conditions, which was also reported in our previous work.^23^

The values of *M*_pCO_2__ and *M*_pH_2_O_ after 1 h of continuous flow at the three RH levels and 400 ppm of CO_2_ are compared for each salt additive in Figure 2a–b. Figure 2a shows that adding salt to the PEI reduces the CO_2_ uptake relative to the salt-free film in a dry atmosphere. We attribute this difference to intermolecular interactions between PEI and the dissociated salt ions that decrease initial zwitterion formation and resulting CO_2_ uptake as carbamate. We hypothesize that the Lewis acid cation associates with the amine lone pair, which inhibits the transfer of electrons to the CO_2_ molecule, and thus hinders zwitterion formation (Scheme S1). This effect is taken in combination with changes to the dry film stiffness (*vide infra*) that contribute to initial CO_2_ uptake by the dry film. The decreased CO_2_ uptake is accompanied by a general increase in the H_2_O uptake as a function of relative humidity in the salt-containing films vs the salt-free film, as shown in Figure 2b. At first, this seems to indicate a simple competitive absorption process between the CO_2_ and H_2_O, but the details of the relationship between PEI, salt, H_2_O, and CO_2_ absorption are more complicated. The H_2_O sorption is not linear with cation identity, indicating that periodic trends within the group are not solely at play either. Therefore, we suggest a mechanism exists wherein initial sorbed species in the dry film (as ammonium carbamate) are protonated to a carbamic acid moiety as the film sorbs H_2_O. We believe that the alkali cation influences both the uptake of water and the equilibrium position favoring carbamic acid/carbamate products. The film with no salt added shows higher initial CO_2_ uptake from the dry film under N_2_, but subsequent uptake at each RH step is not as high. For each sample, the component mass uptake of H_2_O and CO_2_ is directly compared for the different RH experiments in Figure 2c. From these results, we see that the films with salts follow a nonlinear periodic trend with the size of the cation. The CsCl (largest cation) containing sample shows a strong correlation between CO_2_ and H_2_O uptake, where increasing the relative humidity increases both the CO_2_ and H_2_O uptake. However, in the LiCl (smallest cation) containing sample, the two uptakes are not as correlated; increasing the relative humidity from 0% to 20% has almost no effect on CO_2_ content in the film but has some effect on the H_2_O content. The KCl and NaCl salts that have intermediate cation sizes show similar responses that lie between the CsCl and LiCl extremes. Despite some individual correlations, the data for each of the salt-containing films do not fall on a single trendline. This result supports our notion that the mechanism of capture itself is affected by the presence of the salts, presumably influencing the equilibrium point between carbamate and carbamic acid products in humid environments.

**Figure 2:**
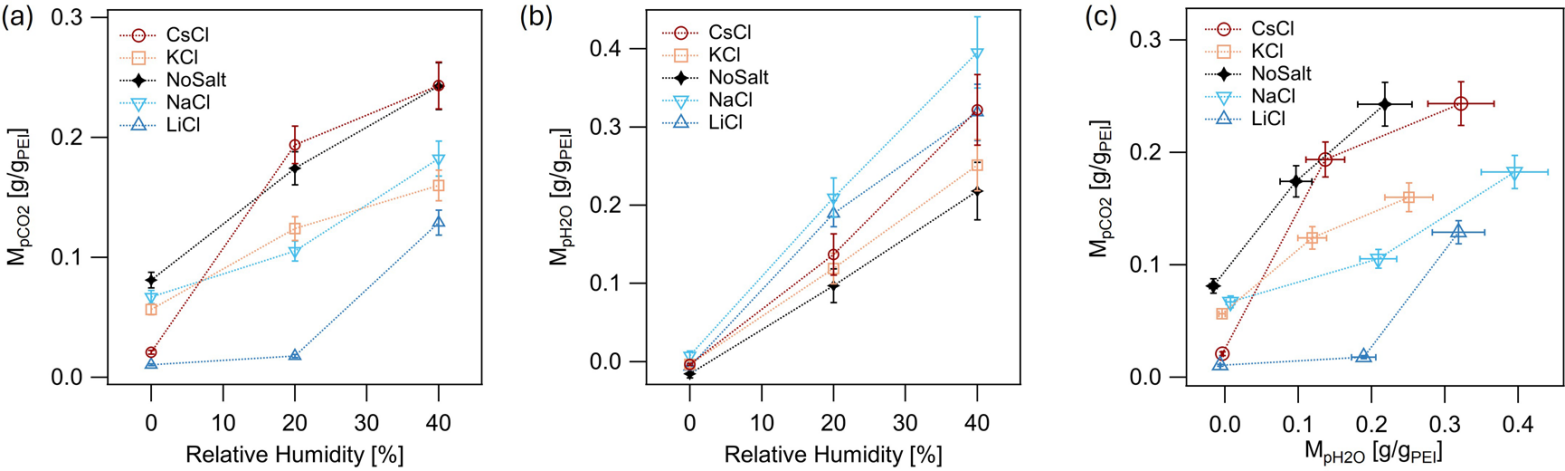
Component uptake for (a) CO_2_ and (b) H_2_O are plotted as a function of RH for each salt added. (c) Direct comparison of *M*_pCO_2__ and *M*_pH_2_O_ reveals that water uptake is not directly responsible for CO_2_ capacity in the salt additive series.

In analyzing this data, we notice that the addition of salt leads to diminished *M*_pCO_2__ in the dry CO_2_ atmosphere. If we account for this initial difference by subtracting the immediate sorption happening in the first 15 min of exposure to dry CO_2_, we can more clearly see the influence of water uptake on carbamate/carbamic acid formation (Figure S2). This analysis method is repeated for later comparison with our ATR-FTIR data discussed in the next section. The difference in *M*_pCO_2__ at the pseudoequilibrium points (at 60, 120, and 180 min) minus *M*_pCO_2__ at 15 min of dry 400 ppm of CO_2_ exposure is presented as a function of both relative humidity and *M*_pH_2_O_ in Figure S2. When this initial CO_2_ capture relative to a N_2_ atmosphere is discounted, we see that the addition of CsCl yields greater CO_2_ capacity than the PEI film with no salt added at nonzero humidities. The positive effect of the cation on CO_2_ uptake is less pronounced as the cation size decreases, with the exception of the LiCl at 40% RH data point, which shows unusual sorption behavior throughout the experiment (Figure 1d). Previous studies have found that LiCl can coordinate with polyamine ligands,^25^ which in aminopolymer sorbents such as PEI, would inhibit water uptake and CO_2_ binding. We propose that a similar phenomenon occurs here, limiting both polymer dynamics and the ability to sorb water and CO_2_. Disregarding the lithium case, our results are in line with the hypothesis that the salt cation influences the carbamate-carbamic acid equilibrium, where larger cations favor the formation of the more amine efficient carbamic acid capture product. We further explore potential origins of these effects by examining the influence of DAC on the mechanical properties of the films.

The QCM-D instrument provides a metric of the mechanical dissipation in the PEI specimens by modeling the peak frequency and width of the resonance peak with a full complex electrical admittance model of the resonator described elsewhere.^26^ The dissipation factor (*D*) reflects the relative stiffness of the film, with a larger *D* indicating a softer, more dissipative film. Here we report the change in dissipation for the *n* = 3 overtone ((15 MHz), Δ*D*_15MHz_), measured between the end point of the 400 ppm of CO_2_ at 0% RH segment (after 60 min exposure) and the specified 20% RH and 40% RH end points (time of 120 and 180 min, respectively) in Figure 3 to quantify the change in dissipation due to sorption of H_2_O in the presence of CO_2_. There is no way to decouple the individual influences of H_2_O and CO_2_ sorption on Δ*D*_15MHz_ in these experiments, but we nevertheless use this analysis to highlight the influence of the different cations on the mechanical behaviors of the films in different environments.

**Figure 3:**
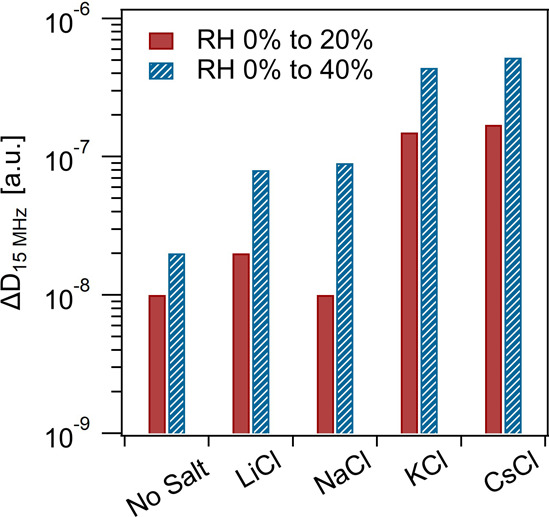
Changes in dissipation due to increases in humidity at 400 ppm of CO_2_ for PEI films in this study.

All films become softer relative to the dry, CO_2_ loaded film as the RH increases and more H_2_O is absorbed into the film, evidenced by the increases in the dissipation factor. However, the magnitude of this change differs depending on the salt added. Comparing the change in end point dissipation after 60 min of 0% RH and 20% RH in 400 pm CO_2_ (red bars in Figure 3), we observe that the larger cation salts (CsCl and KCl) exhibit the largest Δ*D*_15MHz_ by roughly an order of magnitude, indicating the softening effect is more pronounced for these samples. The films containing smaller cation salts (LiCl and NaCl) show smaller degrees of softening at the humidity step from dry to 20% RH, at a level commensurate with the film with no salt added. We believe that the softening may arise from either (i) swelling due to water sorption and/or (ii) shifting CO_2_ capture products from carbamate-ammonium pairs to carbamic acid. Coordination effects between salts and PEI could also contribute to mechanical properties of the films. Referring to the salt series H_2_O uptake results in Figure 2b, we observe that the CsCl and KCl containing samples show lower H_2_O uptake (*M*_pH_2_O_) than NaCl and LiCl samples at 20% RH. Therefore, we do not attribute the observed softening effect at 20% RH purely to sorbed water plasticizing the film, but rather the change in CO_2_ capture product equilibrium, favoring carbamic acid formation. This diminished softening with the addition of CO_2_ in LiCl and KCl doped films relative to KCl and CsCl would be consistent with the previous observation that smaller, stronger Lewis acid cations bias the CO_2_-amine reactions toward carbamate ion formation in the liquid amine adducts.^15,16^ A similar series of dissipation changes is observed at 40% RH (if the LiCl result is neglected), where the smaller cations lead to greater *M*_pH_2_O_ but lesser degrees of softening. The Δ*D*_15MHz_ measured relative to the dry film under N_2_ are reported for each sample at each humidity after 60 min of dosing in Table S1.

Overall, the tandem QCM-D/PM-IRRAS provides estimations of the absolute uptake of the component species in our multicomponent dosing experiments and provides insight into the complexities of intermolecular interactions that underpin sorption capacity. The CO_2_ capacity is directly calculated using a mass to peak intensity relationship determined from dry uptake experiments while the H_2_O mass is derived indirectly by subtracting CO_2_ mass from the total mass. We assert that CO_2_ uptake and mechanical softening effects are driven by carbamic acid formation, as opposed to carbamate-ammonium pair formation, directed by the salt cation identity in humid environments. While advantageous for the wealth of information afforded, each of these evaluations must be conducted in individual experiments that introduce some inherent variability in dosing procedure and sample handling, such as slight differences in relative humidities across the experiment series. In the next section, we describe how the information obtained from tandem QCM-D/PM-IRRAS can be translated to changes in relative carbamate/carbamic acid peak intensities using a high-throughput ATR-FTIR instrument to mitigate these uncertainties.

### ATR-FTIR

In addition to QCM-D/PM-IRRAS, we also employ high-throughput ATR-FTIR spectroscopy to monitor the formation of CO_2_ sorption products and estimate relative H_2_O uptake in PEI films containing the alkali metal chlorides undergoing a single, simultaneous dosing protocol. To accomplish this investigation, a custom gasket-sealed cover with gas/vapor dosing inlets and outlets was engineered to fit over a 24-well array of microgrooved Si wafer ATR crystals (Figure 4a). The plate can be disassembled to release the individual Si wafers for film casting, an example of which is shown in the inset picture in Figure 4a. In one campaign, up to 24 separate samples cast onto the individual ATR crystals were placed in each well with their respective spectra periodically measured in the same 3 h CO_2_/RH dosing profiles used in the QCM/PMIRRAS experiments. The high-throughput ATR-FTIR spectra are acquired serially by rastering the well-plate over a single IR source/detector that measures the sample in the specified well. While the wells are examined individually, the dosing atmosphere is shared among all wells on the plate. For the results presented here, the interval between measurements of the same well is fixed at 2 min (compared to the 1 min intervals for the PM-IRRAS data).

**Figure 4:**
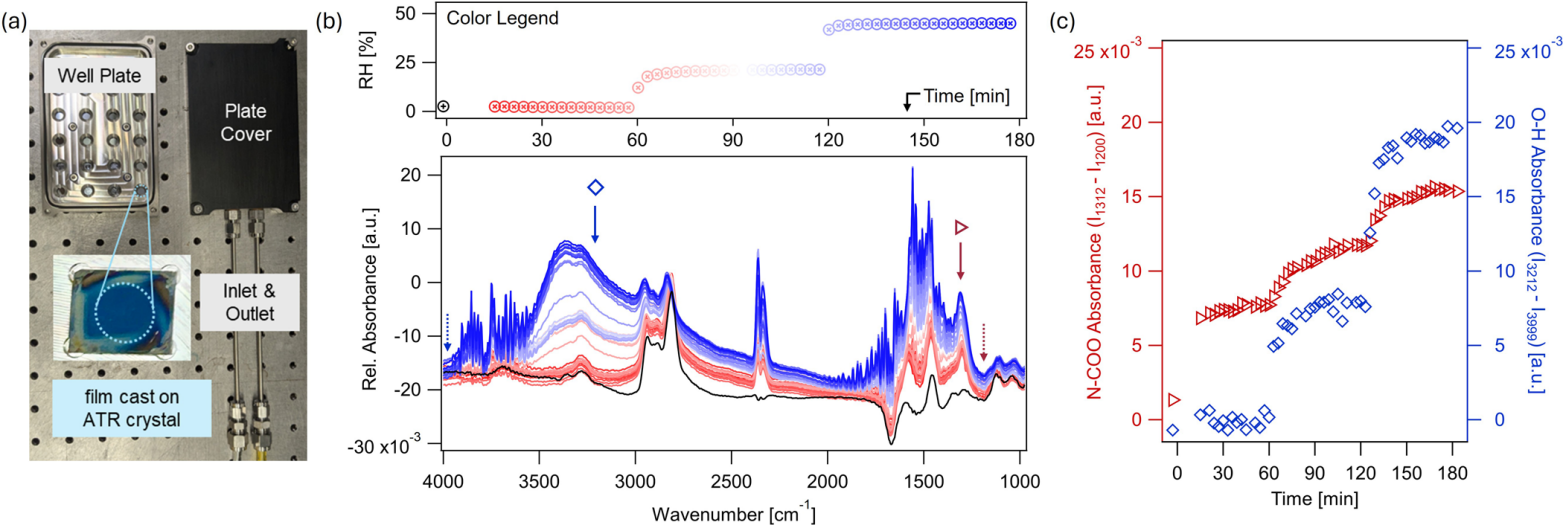
(a) Picture of custom plate, cover with gas/vapor delivery plumbing, and a single coated ATR crystal that is the base of a single well. (b) ATR-FTIR spectra collected as a time series with varying RH at 400 ppm of CO_2_. The black trace is obtained before introduction of CO_2_ while the cell is under N_2_ atmosphere. All traces are relative to a blank reference cell. The top inset depicts the RH color legend for the spectra plotted below. The positions used to calculate the signal and baseline intensities for estimating carbamate/carbamic acid formation (red, triangle) and water sorption (blue, diamond) are denoted by the solid and dashed arrows, respectively. The differences yield a baseline-corrected ATR intensities that are plotted as a function of time in (c).

We depict the spectra and analysis of a single sample (0.1 eq KCl, 100 nm PEI) in one well of one dosing campaign in Figure 4b–c. To estimate the carbamate/carbamic acid formation, the absorbance at 1312 cm^–1^ attributed to the N-COO skeletal vibration^12,27,28^ (noted by the red triangle in Figure 4b) is corrected for baseline increases by subtracting the absorbance at 1200 cm^–1^ (red dashed arrow in Figure 4b) to yield a proxy for CO_2_ capture. This value is plotted versus time in Figure 4c. From our QCM-D/PM-IRRAS experiments, we know that these peak intensities correlate linearly with the uptake mass of CO_2_ measured by QCM-D and assume that this is also a viable approach here. The N-COO skeletal vibration represents the uptake of CO_2_ as both carbamate and carbamic acid within the same general peak position. A similar analysis is performed here to estimate water uptake by subtracting the ATR intensity at 3999 cm^–1^ (blue dashed arrow in Figure 4b), taken as the background, from the intensity at 3212 cm^–1^ (blue diamond in Figure 4b) attributed to a shoulder of the O–H stretching peak that is present in H_2_O (there should be no −OH groups in PEI). We note that spectra collected under humid atmospheres begin to develop fringes observable in the ranges of 3920 cm^–1^ to 3400 cm^–1^ and 2000 cm^–1^ to 1360 cm^–1^ for all experiments. This occurs even in the reference cell that does not contain a PEI film (Figure S3) and is attributed to water vapor. These interference fringes are outside of our wavenumbers of interest chosen for both CO_2_ and H_2_O sorption analysis.

Spectra and analysis of results from a single well are shown in Figure 4b, while we include the spectral series for each individual salt and no salt added films in Figure S4 collected during the same 180 min dosing campaign. The data prior to the first 16 min of dosing is not captured due to current limitations to our experimental setup and analysis workflow. Slight variation in absolute dosing time is noted as each well is scanned individually at some time within the first 2 min of change in gas dosing composition; however, the interval between scans is fixed at 2 min. For comparison of multiple experiments, the change in relative absorbance from the predosed sample under an N_2_ environment (0 min) is measured as a function of time and initialized to a value of 0. Once initialized, the change in absorbance at each well can then be averaged and compared with statistical evaluations as presented in Figure 5a–b for the N-COO and O–H signals, respectively, where nine or more experiments are repeated for each film composition. Error bars depict one standard deviation from the average result at each time point and should be taken to reflect a combination of (i) sample heterogeneity, (ii) variation in dosing time within the 2 min iterative sampling interval, and (iii) slight variation in plate conditioning and dosing conditions that should yield a common experimental uncertainty across the samples measured in the same campaigns.

**Figure 5:**
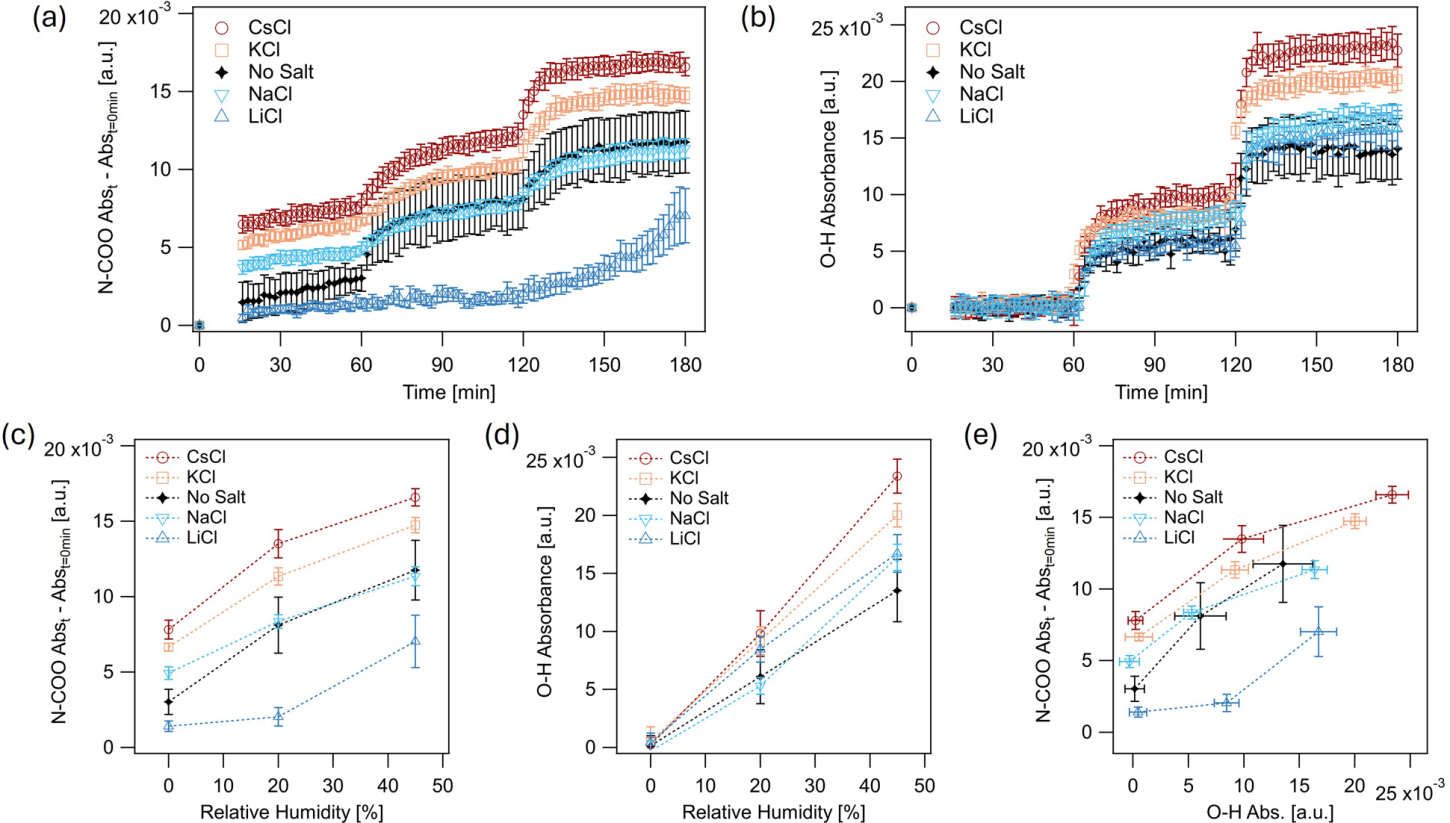
Average relative (a) N-COO and (b) O–H signals (*n* ≥ 9) for PEI systems investigated as a function of dosing with high-throughput ATR-FTIR initialized by the absorbance measured under dry N_2_ (prior to dosing, *t* = 0 min). The average absorbance after 60 min of exposure in each dosing condition are summarized in panels (c) and (d) for the N-COO and O–H signals, respectively. The relationship between measured N-COO and O–H signals are assessed in (e). Error bars represent one standard deviation, lines are included to guide the eye.

The average N-COO and O–H absorbances at the end of each 60 min dosing step are summarized in Figure 5c–e for further analysis. The increases in average N-COO absorbance upon salt incorporation clearly indicate that the salts influence CO_2_ uptake behavior in humid environments, with the CsCl additive displaying the greatest increase in CO_2_ and H_2_O sorption. The results in Figure 5c show a positive correlation between the larger cation radius of the alkali metal chloride and the relative N-COO signal for the salt series, suggesting that adding cations with increased polarizability may be attributed to increased CO_2_ capture. The profile for the film containing LiCl shows a lack of apparent steps with changing dosing conditions and fails to reach a pseudoequilibrium plateau in N-COO absorbance at a relative humidity of 45%, mirroring the result from QCM-D/PM-IRRAS experiments (Figure 1d). While LiCl still follows periodic trends with the lowest increase in relative N-COO signal, its effect is adverse, and compared to PEI films without salt, PEI films with LiCl lead to less CO_2_ sorption. The KCl and NaCl salts in the middle of the series fall between the two extremes of the CsCl and LiCl containing samples.

In addition to the carbamate/carbamic acid signal, the relative O–H stretching signal was used to estimate water uptake. Figure 5d shows that the no salt added samples had, on average, the lowest relative water uptake, and that the addition of salts consistently led to increased water uptake. The increase in estimated water sorption appears to follow the CO_2_ uptake trends, with the most polarizable cations having the highest relative water signal; however, the uncertainty in these values is significantly larger than for CO_2_ uptake. There could be a convolution of the water signal with carbamic acid O–H stretching, leading to an inflated estimation of water uptake in this interpretation method. A better attribution of this signal change is therefore a sum of H_2_O and carbamic acid contributions, rather than just H_2_O uptake. Figure 5e plots the relationships between average N-COO and O–H signals at the end point of each dosing segment for the salt series. As was observed in QCM-D/PM-IRRAS results (Figure 2c), the effect of the salt generally increases the positive relationship between presumed water sorption (O–H signal) and CO_2_ uptake (N-COO signal), but there is not a common trendline among all traces. The larger cations (Cs^+^ and K^+^), on average, show increased N-COO signals than the PEI sample without salt added at all RH steps, while the smaller cations (Na^+^ and Li^+^) yield equivalent or less uptake. The difference in effect of cation identity on the N-COO vs O–H relationship strengthens our argument that the cation is exerting influence on the carbamate/carbamic acid formation equilibrium, rather than just increasing water sorption, that influences overall CO_2_ capture.

In Figure 5e, differences in N-COO signal at the end of the dry CO_2_ exposure segment (at 60 min of dosing in the campaign profile) offset the samples and complicate interpretation of water’s influence on CO_2_ sorption. Similar to our QCM-D/PM-IRRAS analysis above, we initialize to the N-COO ATR-FTIR intensity after 16 min of dosing rather than the initial 0 min intensity to account for differences in CO_2_ uptake in the dry state in Figure S2. The trends in the resulting profiles are quite similar, despite differences in the data collection and analysis methods, between the two approaches. To summarize approaches from the two instrument platforms, the QCM-D/PM-IRRAS data estimate a CO_2_ mass that is then subtracted from total mass uptake to indirectly yield a H_2_O component mass, while ATR-FTIR uses a direct change in peak intensity that represents, at least in part, the increase in H_2_O sorption. The possible convolution of the water O–H stretching vibration with that of carbamic acid’s O–H band complicates the estimation of H_2_O, however. In the ATR-FTIR results initialized to the average intensity at 16 min (Figure S2), the correlation between the N-COO and O–H peak intensities again do not fall on a single trendline, supporting the notion that the mechanism of capture is affected by the presence of salt additives and are therefore capable of significantly influencing the CO_2_ capacity and water sorption tendencies of the PEI films. Agreement in relative trends between the two measurement platforms and analysis methods demonstrates the promise of the high-throughput ATR-FTIR for future studies in DAC sorbent development and exploration of structure–property-relationships in such materials.

## Conclusion

Herein, we present an analysis of DAC performance obtained by PEI films with added LiCl, NaCl, KCl, or CsCl salts measured by two distinct instrument platforms, a tandem QCM-D/PM-IRRAS and high-throughput ATR-FTIR to understand sorption behavior in the modulated PEI films. The tandem QCM-D/PM-IRRAS measured both the peak intensities and mass uptake of the PEI films concurrently, allowing us to obtain an absolute quantity of each component sorbed by the PEI films. We observed an increase in the CO_2_ sorbed in the PEI films with the addition of CsCl, but either no difference or a decrease in CO_2_ sorption relative to a fully desorbed PEI film with the addition of the other group 1 chloride salts. When the data is initialized to account for differences in uptake of CO_2_ under dry conditions, the effect is more pronounced and indicates that water plays a major role in determining the uptake of CO_2_ in PEI. Direct comparison of water and CO_2_ uptake trends reveals that the different salt additives impart different, nonlinear influences on intermolecular PEI-CO_2_–H_2_O interactions. We learn more about these interactions by examining changes in film stiffness via the QCM dissipation factor, which shows the heavier cations promote more film softening with less water uptake than the lighter salts or salt-free PEI samples. We conclude that the larger cations favor formation of carbamic acid over carbamate-ammonium capture products, leading to increases in CO_2_ capture and decreases in intermolecular cross-linking that contribute to stiffening.

High-throughput ATR-FTIR spectroscopy was utilized to estimate the formation of carbamate/carbamic acid species and water uptake through absorbance peaks at characteristic wavenumbers corresponding to N-COO and O–H bands for large numbers of films. The addition of alkali-salts was seen to increase the CO_2_ adsorption following periodic trends. Inclusion of highly polarizable Cs^+^ ions was seen to have the greatest increase in N-COO bond formation relative to samples without salts, while the addition of Li^+^ ions had an adverse effect. We find that all salts examined had a positive effect on water sorption based on our analysis, but caution that our approach may be confounded by other O–H containing species formed concomitantly with water sorption (such as carbamic acid).

While the PEI film without the addition of the chloride salts lies at different points in the sample series for CO_2_ uptake for both QCM-D/PM-IRRAS and ATR-FTIR data, films containing salt followed similar trends across results from both instruments. The PEI films with salt followed a periodic trend based on cation identity, where the CO_2_ uptake increased with increasing ionic radius and increasing polarizability of the ion. We believe the consistency in this trend uncovers the intricate role of cation-CO_2_ interactions in the PEI films, where the addition of the ionic salts biases the CO_2_ capture equilibrium depending on the cation identity. The results and analysis we present here will be useful for exploring further applications of amine-containing polymers in the DAC of CO_2_, where both the process environment and material optimization are crucial, and small amounts of inexpensive additives can influence performance outcomes. Increased water uptake may not be desirable in all environments, as desorption of water is energetically costly, however insight into the fundamental nature of the intermolecular interactions dictating sorbent reactivity can be valuable. Absolute uptake quantitation with QCM-D/PM-IRRAS alongside robust and automatable ATR-FTIR experiments are valuable tools to explore this landscape of intermolecular interactions influencing a sorbent’s DAC potential. In the future, it would be advantageous to further research the effect of other salts with larger, more polarizable molecular cations on amine-bearing polymers and their DAC abilities, as well as explore thermodynamic implications of salt additives on sorption/desorption profiles.

## Supplementary Material



## Notes

Additional schemes, PM-IRRAS/QCM-D uptake profiles, data analysis, and ATR-FTIR spectra (PDF)
