# Supplementary material for: Investigating the Influence of Alkali Chloride Salts and Hydration on the Direct Air Capture Capacity of Polyethylenimine Films with Quartz Crystal Microbalance and Infrared Spectroscopy
Source: Langmuir. 2026 Jun 3;42(23):16170–9. doi: 10.1021/acs.langmuir.6c00429 (PMC13276903; doi:10.1021/acs.langmuir.6c00429)
Supplement: Supplementary file 1 [file la6c00429_si_001.pdf]

Supporting Information for

Investigating the Influence of Alkali Chloride Salts and Hydration on the Direct Air Capture Capacity of Polyethyleneimine Films with Quartz Crystal Microbalance and Infrared Spectroscopy

Kayley Winata[1†], Kayli Kuk[2†], Christopher L. Soles[3], Avery E. Baumann[3*]

[1]Portola High School, Irvine, CA 92618, USA

[2]Northwest High School, Germantown, MD 20874, USA

[3]Materials Science and Engineering Division, National Institute of Standards and Technology, Gaithersburg, MD 20899, USA

[†]Authors contributed equally

[*]Corresponding author, avery.baumann@nist.gov

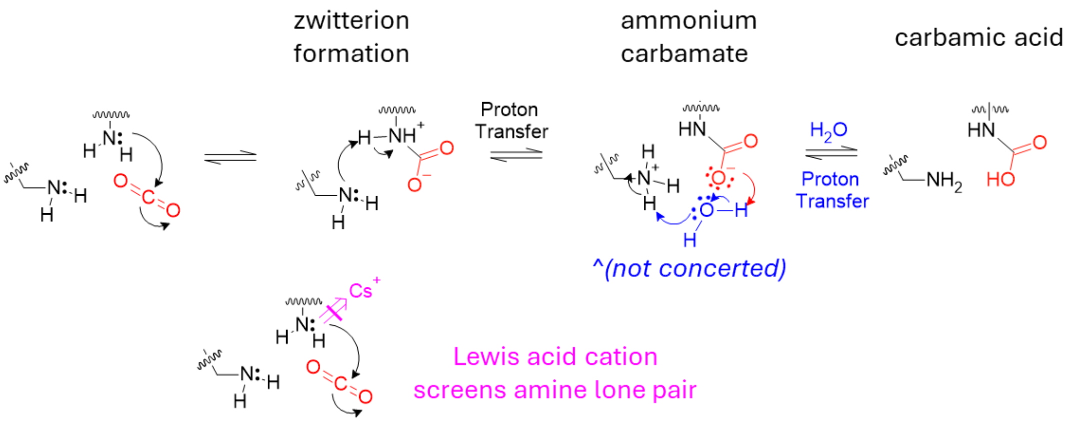

Scheme S1. The proposed mechanism of capture is influenced by the Lewis acid cation withdrawing electron density from the amine lone pair electrons. The lone pair electrons are transferred to the CO_2 carbon in the zwitterion formation step. The Lewis acid cation may similarly influence water-mediated proton transfers occurring along the reaction pathway of ammonium carbamate to carbamic acid (not explicitly shown).

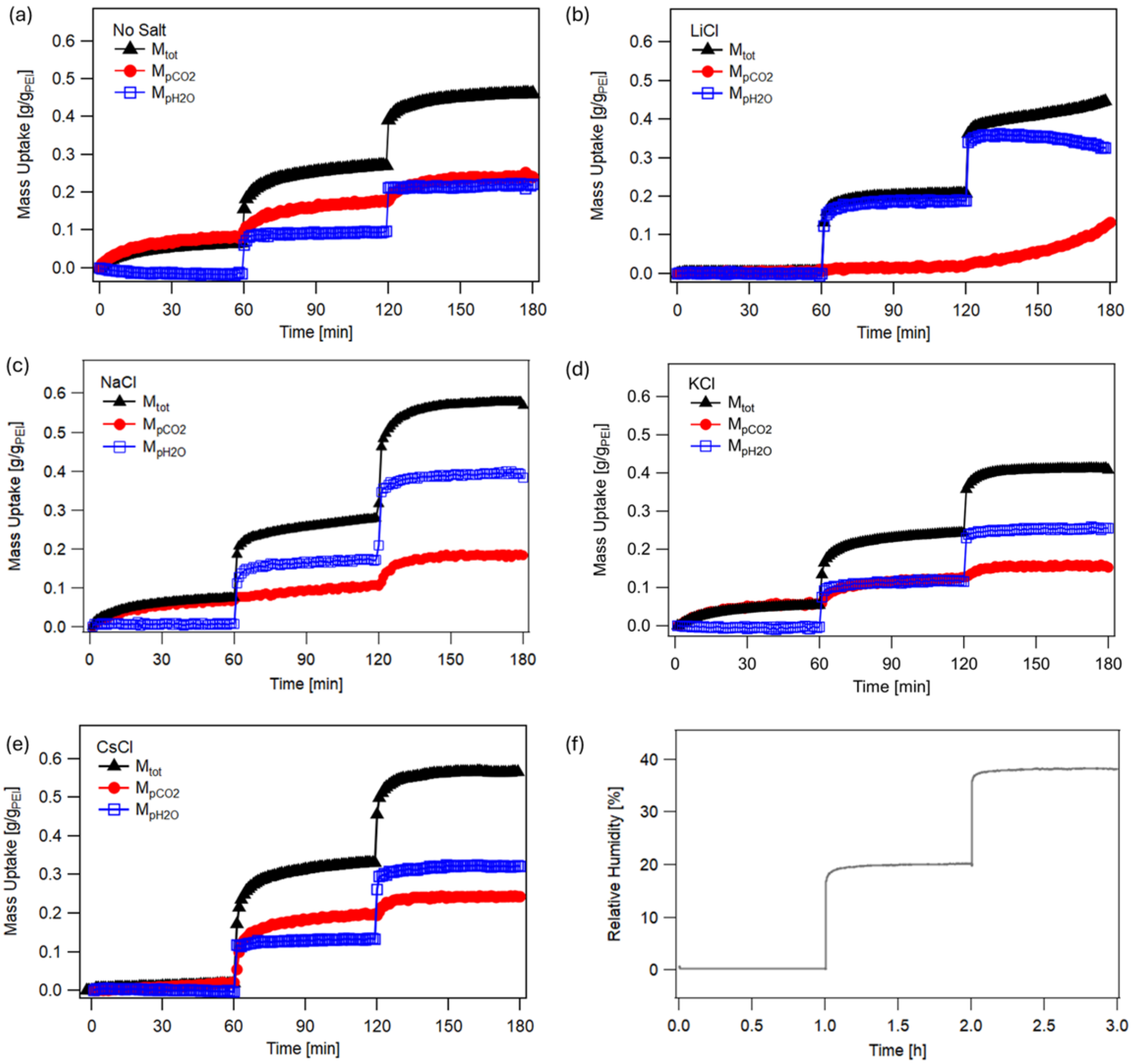

Figure S1. Full QCM-D/PM-IRRAS uptake profiles for all PEI samples (a) without and (b-e) with salt additives examined in this study. The data are shown for (b) LiCl, (c) NaCl, (d) KCl, and (e) CsCl salt additives. A representative RH profiles is included in panel (f).

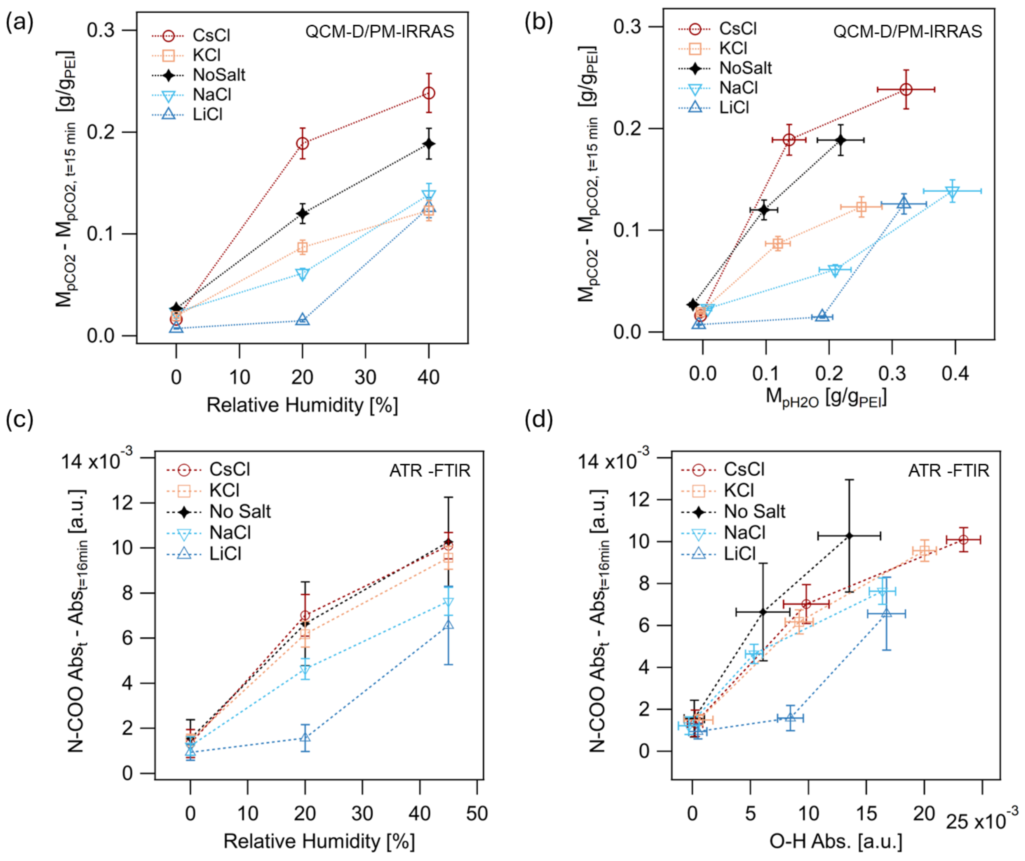

Figure S2. Comparison of QCM-D/PM-IRRAS (top) and ATR-FTIR (bottom) summary plots describing CO_2 uptake trends as a function of relative humidity (a, c) and the proxy for sorbed water for each analysis method (b, d). The uptake with increasing humidity/water for each salt series appear to follow a similar shaped path for each of the two measurement methods, however order within the salt series varies slightly.

Table S1. Changes in dissipation for the n = 3 overtone from a dry film state under N_2 to exposure to 400 ppm at the designated RH % after 60 min dosing. A negative number indicates film stiffening relative to the film at the dry state under N_2.

	ΔD_{15MHz} from dry N_2 state		
	0 % RH	20 % RH	40 % RH
No Salt	-6.8E-09	1.1E-07	1.9E-07
LiCl	1.23E-07	3.8E-07	1.54E-06
NaCl	-6.4E-09	1.4E-07	2.9E-07
KCl	-3.9E-08	-2E-08	-1E-08
CsCl	-2.2E-08	-3.2E-08	-1.2E-08

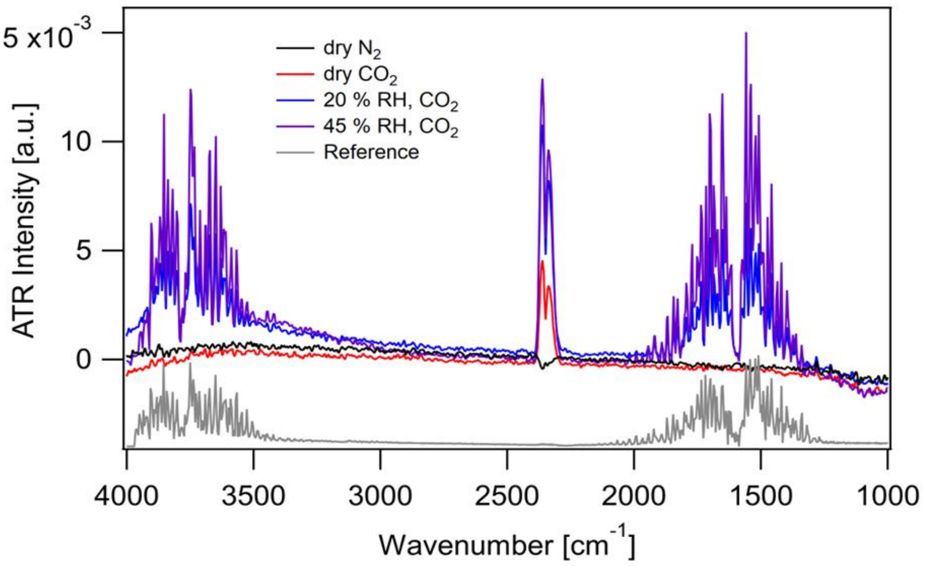

Figure S3. ATR-FTIR spectra from an uncoated reference well in the plate during the same dosing campaign as the data presented in Figure 4 and Figure S4. The reference spectrum is of gas phase water.[S1]

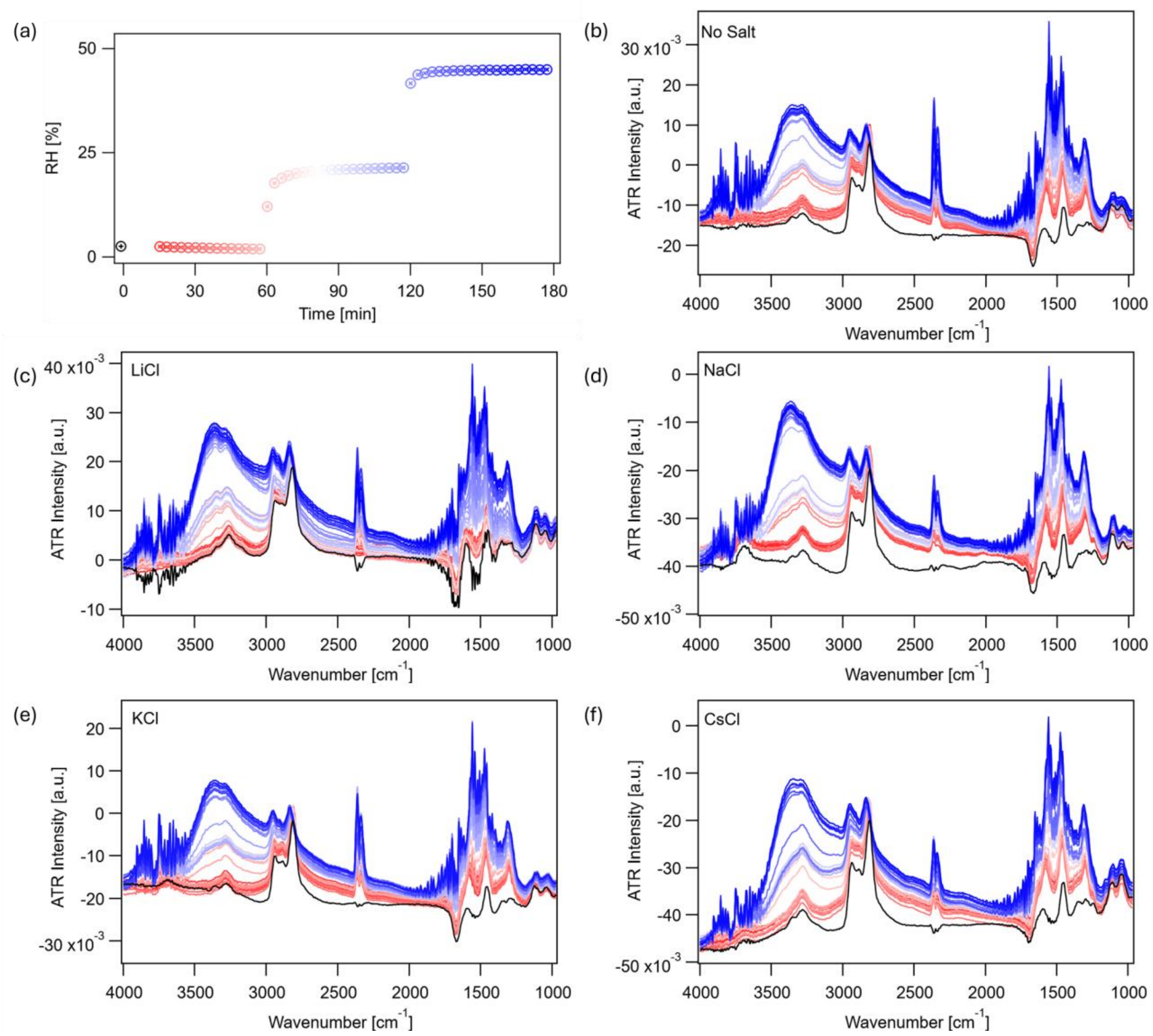

Figure S4. (a) The RH profile obtained during the multi-sample experiment run corresponding to the color legend spectra in panels (b-f). The plot serves as a colorimetric legend for the subsequent panels. Waterfall plots showing the spectra collected for one ATR-FTIR well containing a 100 nm PEI film with (b) no salt, (c) LiCl, (d) NaCl, (e) KCl, or (f) CsCl added. All samples were measured in 3 min intervals during the same 180 min dosing period.

References

(S1) NIST Mass Spectrometry Data Center, William E. Wallace, director, "Infrared Spectra" in NIST Chemistry WebBook, NIST Standard Reference Database Number 69, Eds. P.J. Linstrom and W.G. Mallard, National Institute of Standards and Technology, Gaithersburg MD, 20899, https://doi.org/10.18434/T4D303, (retrieved November 17, 2025).
